# Social capital is associated with lower mosquito vector indices: secondary analysis from a cluster randomised controlled trial of community mobilisation for dengue prevention in Mexico

**DOI:** 10.1186/s12963-019-0199-3

**Published:** 2019-12-10

**Authors:** Víctor Alvarado-Castro, Sergio Paredes-Solís, Elizabeth Nava-Aguilera, Arcadio Morales-Pérez, Miguel Flores-Moreno, José Legorreta-Soberanis, Esmeralda Jaimes-Néstor, Anne Cockcroft, Neil Andersson

**Affiliations:** 1 Centro de Investigación de Enfermedades Tropicales (CIET), Universidad Autónoma de Guerrero, Calle Pino s/n. Colonia El Roble, CP, 39640 Acapulco, Guerrero México; 2 Department of Family Medicine, McGill University, Montreal, Canada

**Keywords:** Social capital, Community mobilisation, Dengue, Factor analysis, Vector indices

## Abstract

**Background:**

Control of the *Aedes aegypti* mosquito is central to reducing the risk of dengue, zika, chikungunya, and yellow fever. Randomised controlled trials, including the *Camino Verde* trial in Mexico and Nicaragua, demonstrate the convincing impact of community mobilisation interventions on vector indices. These interventions might work through building social capital but little is known about the relationship between social capital and vector indices.

**Methods:**

A secondary analysis used data collected from 45 intervention clusters and 45 control clusters in the impact survey of the Mexican arm of the *Camino Verde* cluster randomised controlled trial. Factor analysis combined responses to questions about aspects of social capital to create a social capital index with four constructs, their weighted averages then combined into a single scale. We categorised households as having high or low social capital based on their score on this scale. We examined associations between social capital and larval and pupal vector indices, taking account of the effects of other variables in a multivariate analysis. We report associations as odds ratios and 95% confidence intervals.

**Results:**

The four social capital constructs were *involvement*, *participation*, *investment*, and *communication*. Among the 10,112 households, those in rural communities were much more likely to have a high social capital score (OR 4.51, 95% CIca 3.26–6.26). Households in intervention sites had higher social capital, although the association was not significant at the 5% level. Households with high social capital were more likely to be negative for larvae or pupae (OR 1.38, 95% CIca 1.12–1.69) and for pupae specifically (OR 1.37, 95% CIca 1.08–1.74). There was interaction between intervention status and social capital; in multivariate analysis, a combined variable of intervention/high social capital remained associated with larvae or pupae (ORa l.56, 95% CIca 1.19–2.04) and with pupae specifically (ORa 1.65, 95% CIca 1.20–2.28).

**Conclusion:**

This is the first report of an association of high social capital with low vector indices. Our findings support the idea that the *Camino Verde* community mobilisation intervention worked partly through an interaction with social capital. Understanding such interactions may help to maximise the impact of future community mobilisation interventions.

## Background

Social capital is a concept now widely used in sociology, economics, education, and more recently in epidemiology [1]. It includes real or potential resources, social structures, and regulated interactions between them [2, 3]. There is no agreed definition, but a common definition is “the set of characteristics of social organisation, such as confidence, norms, and networks that may improve the effectiveness of society by facilitating coordinated actions” [4]. Social capital represents the social connections and benefits generated by them and is associated with values that reinforce social cohesion, such as tolerance, solidarity, and confidence [5]. Most definitions focus on social relationships that have productive benefits. Many instruments have been used to measure social capital [6–8]. The components of social capital measured by these instruments include personal relationships, social support networks, participation and confidence of citizens, and rules for cooperation [5–7, 9].

Social capital may be important in the health of a population. Although mechanisms are unclear, authors have reported positive associations with physical and mental health [10–12]. High social capital has been linked to reduced crime, drug use, and alcoholism [13, 14]. In communities with low social capital, inhabitants report increased levels of stress [15], the well-being of children and elderly people is lower [16, 17], and the ability to respond to environmental health risks is reduced [18]. All this suggests that measuring social capital could be useful in epidemiological studies of population health [19–21].

The World Health Organization recommends control of the *Aedes aegypti* vector as the mainstay of efforts to prevent yellow fever [22], dengue [23], zika, and chikungunya [24], despite an existing vaccine for yellow fever [25] and recent advances in developing a vaccine for dengue [26, 27]. Several trials have measured the impact of community mobilisation for control of the dengue vector [28–33]. A recent systematic review concluded that community mobilisation was effective in reducing vector indices [34]. Community mobilisation strategies for dengue prevention may work, at least in part, by strengthening existing bonds between members of the community and increasing their level of social capital [35]. One small study examined the correlation between some social capital elements and vector indices [36], but we have found no other published reports of attempts to relate measures of social capital to vector indices. We used data from the impact survey of the *Camino Verde* cluster randomised control trial of evidence-based community mobilisation for dengue prevention in Mexico and Nicaragua [33] to examine the association of a measure of social capital with vector indices. We also explored how social capital and its association with vector indices were affected by the trial community mobilisation intervention, to examine the possibility that the lower vector indices in intervention sites might be mediated, at least in part, through an interaction between the intervention and social capital.

## Methods

Details of the methods and findings of the Camino Verde trial are described in detail elsewhere [33]. In brief, the trial involved using evidence-based discussions to stimulate communities to design their own strategies for non-chemical control of the *Aedes aegypti* vector in their communities. The Mexican arm of the trial included 45 intervention clusters and 45 control clusters. The trial impact survey took place in all 90 clusters in late 2012 and included a household questionnaire survey, administered by trained interviewers to one member per household, and a household entomological survey. We used data from the household survey to construct a social capital index and data from the entomological survey to calculate vector indices for each household.

### Indicator of social capital

Our social capital index was based on 21 questions from the household survey, initially categorised according to the four domains of social capital proposed by Siegler [5]: personal relationships, social network support, civic engagement, and trust and cooperative norms (Table 1). The response to each question was dichotomous, mostly Yes or No. The way we categorised the possible responses for other questions is shown in Additional file 1: Table S1.

**Table 1:** Questions and labels included in the factor analysis for the social capital index

Dimension	Question	Label
Personal relationships	P27. How much do you talk with your family members about how to avoid mosquitoes in the house: a lot, a little or not at all?	Family communication
	P28. How much do you talk to your neighbours about how to avoid mosquitoes in the neighbourhood: a lot, a little or not at all?	Neighbourly communication
Social network support	P7. Do the neighbours in this street help each other?	Mutual assistance
	P8. When a family in the community has a wedding, who helps with the preparations for the wedding?	Festive help
	P9. When a family in the community has a death, who helps with the wake, burial and prayers?	Grief support
	P12. If your home was destroyed by an earthquake, hurricane or flood, who would give you shelter for at least two weeks?	Disposition
	P26. Who is the most responsible for control of dengue mosquito breeding sites: yourselves, health personnel, or both?	Responsibility
	P32. Who is/are the person or people who work/s most for the health of the people of the community?	Health collaboration
Civic engagement	P10. Would you dedicate part of your time to a project that does not benefit you directly but has benefits for other people in the community?	Solidarity
	P6. When there is a problem in the community, who decides about its solution?	Self-Management
	P11. Do you think your neighbour would dedicate part of his/her time to a project that does not directly benefit him/her but does benefit other people in the community?	Confidence
	P17. Does anyone in this house participate in a group or association?	Social participation
	P18. In the last year, has anyone from this household participated in a parade or meeting related to health?	Health participation
	P29. So far this year, how many times have you met in the community to talk about how to avoid mosquitoes?	Community meeting
	P31. How many people in the house have participated with the people of the community, in activities to control mosquitoes?	Dengue participation
Trust and cooperative norms	P21. In your opinion, has the mistreatment of women in this community increased, decreased or remained the same?	Friendliness
	P22. Do you feel safe in your community or neighbourhood?	Safety
	P4. Do you consider that this community can avoid dengue on its own?	Self-sufficiency
	P22. Do you think it is worth spending time and money each week to eliminate mosquito breeding sites in your home?	Individual benefit
	P23. Do you think your neighbours feel it is worth spending time and money each week to eliminate mosquito breeding sites in their homes?	Collective benefit
	P30. What is the main activity that has given the best results to the people of this community for the control of mosquitoes?	Identified activity

Factor analysis requires that there be no missing values for responses to any of the included questions. Among the 21 questions, the highest proportion of missing data was 1.9%. We used Amelia II [37] to impute the values of the missing data with an expectation-maximisation algorithm for the binary variables, conciliating the data from 10 series of imputed data using the Rubin method [38].

We undertook a factor analysis to determine the weights of the individual variables in an overall social capital index and the domains (constructs) within the index, using the “psych” package in R [39, 40]. We created a scree plot of eigenvalues, used an eigenvalue of 1.15 as the cut-off for inclusion in the final index, and carried out a parallel analysis with 100 simulations to decide which factors should remain in the final index. We assumed that all the variables would be correlated and we used oblique rotation to group the retained elements. We then dichotomised the social capital score of each household as high or low, based on the frequency distribution of the social capital scores.

### Vector indices

For this analysis, we calculated four *Aedes aegypti* indices at household level:

1. Household positivity for larvae or pupae. We categorised a household as positive for larvae and/or pupae if any of the containers inspected in the household contained any larvae or pupae.
2. Household positivity for pupae. We categorised a household as positive for pupae if any container inspected contained any pupae. Pupal indices may be better predictors of the adult population of mosquitoes [41, 42].
3. Pupae per household index (PHI). Number of pupae found per household
4. Pupae per person index (PPI). Number of pupae per household divided by the number of household members.

### Analysis of associations

Analysis of associations between social capital, intervention status, and vector indices relied on CIETmap, an open source interface with the R programming language [43]. We used the Mantel-Haenszel procedure [44] to examine associations, and the Zelen test for heterogeneity to test for the significance of interactions between variables [45], conducting first bivariate and then multivariate analysis of variables associated with larvae/pupae and with pupae alone. Initial saturated multivariate models included those variables significantly associated with the outcome in bivariate analysis. We express associations as odds ratios (OR) and 95% confidence intervals, adjusted for clustering (95% CIca) by the Lamothe method [46].

## Results

A total of 10,112 households (5181 in intervention clusters and 4931 in control clusters) provided responses to the household questionnaire and entomological data. The average percentage of missing data for the 21 variables to be included in the factor analysis for the social capital index was 0.42% among households in the intervention sites and 0.39% among households in control sites.

### Social capital score

**Fig. 1:**
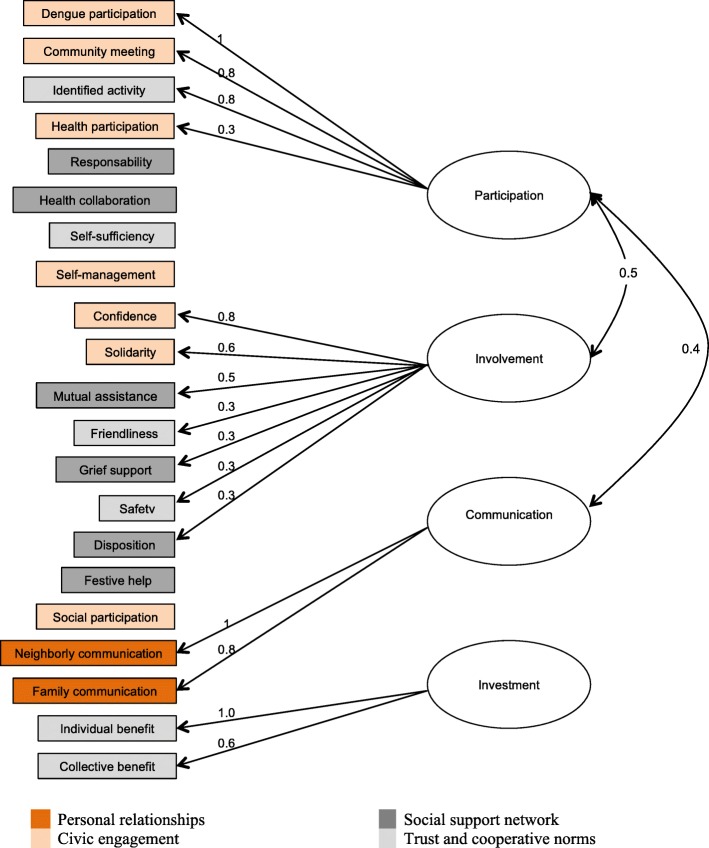
Path plot of the oblique rotation of the constructs in the social capital index. Root mean square residual (RMSR) = 0.04, root mean square error of approximation (RMSEA) = 0.067, 90% CI 0.065–0.068

The factor analysis produced a social capital scale from four constructs that we interpreted as involvement, participation, investment, and communication. Figure 1 shows the combinations of the individual dichotomous variables into the four constructs. As shown in Fig. 1, some of the original variables did not have significant weight in any of the four constructs. Table 2 shows the weights of each variable in the overall social capital index and in the four constructs. Each construct groups variables with a weight of 0.3 or above for that construct. Half (50%) of the entire variance of the social capital index was explained by the four constructs, with 35% being explained by two of the constructs: participation and involvement.

**Table 2:** Weights of the individual variables in each of the four constructs

Variables	Weights of the variables in each construct			
	Participation	Involvement	Communication	Investment
Dengue participation	*0.98*	− 0.06	0.01	0.08
Community meeting	*0.83*	0	0.08	− 0.02
Identified activity	*0.77*	− 0.11	− 0.08	− 0.01
Health participation	*0.33*	0.27	0.05	− 0.08
Responsibility	0.24	0.02	0.01	− 0.14
Health collaboration	0.21	− 0.04	0	0.06
Self-sufficiency	0.16	0.04	0.03	0.01
Self-management	− 0.09	− 0.02	− 0.01	− 0.03
Confidence	− 0.14	*0.78*	− 0.02	− 0.01
Solidarity	0.02	*0.62*	− 0.04	0.05
Mutual assistance	0	*0.52*	0.02	0.05
Friendliness	0.04	*0.33*	0.02	− 0.01
Grief support	− 0.05	*0.33*	0	− 0.06
Safety	0.11	*0.32*	− 0.02	− 0.05
Disposition	− 0.08	*0.3*	0.05	− 0.02
Festive help	0.01	*0.3*	− 0.03	− 0.01
Social participation	0.03	0.21	0.02	− 0.03
Neighbourly communication	0.06	− 0.01	*0.98*	− 0.08
Family communication	− 0.03	− 0.06	*0.76*	0.02
Individual benefit	0.06	− 0.01	− 0.09	*1*
Collective benefit	0.02	0.1	0.1	*0.64*
% Variance of the index	22.3	12.2	8.0	7.2
Eigenvalue	2.82	1.48	1.43	1.17

**Fig. 2:**
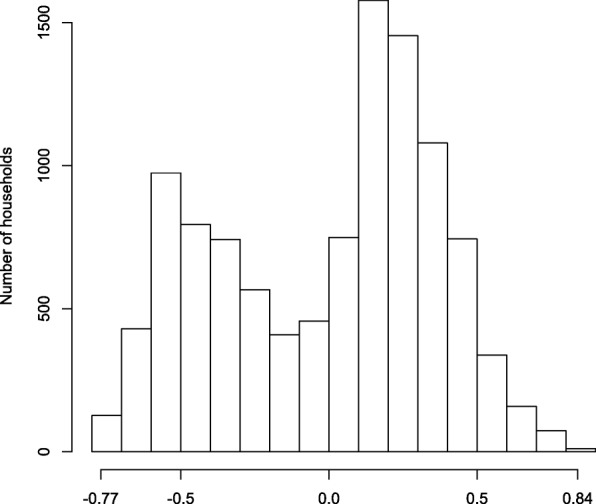
Frequency distribution of household social capital scores

The social capital index, calculated as a weighted average of the four constructs, had a minimum value of − 0.77 and a maximum value of 0.84. Figure 2 shows the frequency distribution of the social capital index among all households; the bimodal distribution was similar between intervention and control households. For the analysis of social capital in relation to other variables, we dichotomised the scores as below 0 (low social capital) and 0 or above (high social capital).

Households in rural areas were much more likely to have a high social capital score than households in urban areas (58% vs 23%, OR 4.51, 95% CIca 3.26–6.26). Households in intervention clusters were more likely to have a high social capitals score than those in control clusters, although this difference was not significant at the 5% level (OR 1.31, 95% CIca 0.87–1.98).

### Social capital and vector indices

Tables 3 and 4 show bivariate associations with presence of larvae and/or pupae and with presence of pupae specifically. Households with a high social capital score were significantly more likely to be negative for larvae and/or pupae, and more likely to be negative for pupae specifically. The association between social capital and vector indices was stable across four different constructions of the social capital index and categorisation into high and low social capital. Details of these sensitivity analyses are in Additional file 1.

**Table 3:** Bivariate associations with absence of larvae or pupae in households

Variable	Level	*N* (%) households with larvae or pupae		OR (95% CIca)
		Absent	Present	
In intervention community	Yes	4543 (87.7)	638 (12.3)	*1.60 (1.12–2.27)*
	No	4028 (81.7)	903 (18.3)	
Household social capital score	High	3763 (87.1)	558 (12.9)	*1.38 (1.12–1.69)*
	Low	4808 (83.0)	983 (17)	
Intervention and high social capital	Yes	2153 (90.4)	229 (9.6)	*1.92 (1.37–2.69)*
	No	6418 (83.0)	1312 (17.0)	
Type of community	Rural	4948 (85.8)	822 (14.2)	1.19 (0.83–1.72)
	Urban	3623 (83.4)	719 (16.4)	
House construction	Temporary	3384 (85.0)	597 (15.0)	1.03 (0.85–1.24)
	Permanent	5130 (84.6)	931 (15.4)	
Receive govt social programme	Yes	4305 (86.4)	676 (13.6)	*1.29 (1.05–1.59)*
	No	4244 (83.1)	861 (16.9)	
Education of household head	Low	3371 (83.7)	656 (16.3)	0.88 (0.76–1.01)
	Higher	5117 (85.4)	876 (14.6)	
Temephos in household water	No	6613 (83.0)	1355 (17.0)	*0.46 (0.36–0.60)*
	Yes	1958 (91.3)	186 (8.7)	

*Italicised font* indicates associations significant at the 5% level

*OR* odds ratio, *CIca* cluster adjusted confidence intervals

**Table 4:** Bivariate associations with absence of pupae only in households

Variable	Level	*N* (%) households with pupae		OR (95% CIca)
		Absent	Present	
In intervention community	Yes	4857 (93.7)	324 (6.3)	*1.68 (1.13–2.52)*
	No	4433 (89.9)	498 (10.1)	
Household social capital score	High	4026 (93.2)	295 (6.8)	*1.37 (1.08–1.74)*
	Low	5264 (90.9)	527 (9.1)	
Intervention and high social capital	Yes	2267 (95.2)	115 (4.8)	*1.98 (1.34–2.94)*
	No	7023 (90.9)	707 (9.1)	
Type of community	Rural	5325 (92.3)	445 (7.7)	1.14 (0.75–1.73)
	Urban	3965 (91.3)	377 (8.7)	
House construction	Temporary	3661 (92.0)	320 (8.0)	1.02 (0.83–1.26)
	Permanent	5563(91.8)	498 (8.2)	
Receive govt social programme	Yes	4612 (92.6)	369 (7.4)	1.21 (0.94–1.55)
	No	4655 (91.2)	450 (8.8)	
Education of household head	Low	3637 (90.3)	390 (9.7)	*0.72 (0.60–0.86)*
	Higher	5564 (92.8)	429 (7.2)	
Temephos in household water	No	7234 (90.8)	734 (9.2)	*0.42 (0.30–0.59)*
	Yes	2056 (95.9)	88 (4.1)	

*Italicised font* indicates associations significant at the 5% level

*OR* odds ratio, *CIca* cluster adjusted confidence intervals

Other variables significantly associated with the vector indices were location (urban or rural), type of house construction (permanent vs non-permanent), education of the household head, coverage with a government social programme, and presence of temephos in household water containers. There was an interaction between social capital and intervention status in their association with vector indices, and a composite variable (1 = with intervention and with high social capital, 2 = either no intervention or low social capital) was strongly associated with absence of the larvae/pupae and absence of pupae alone (Tables 3 and 4).

**Fig. 3:**
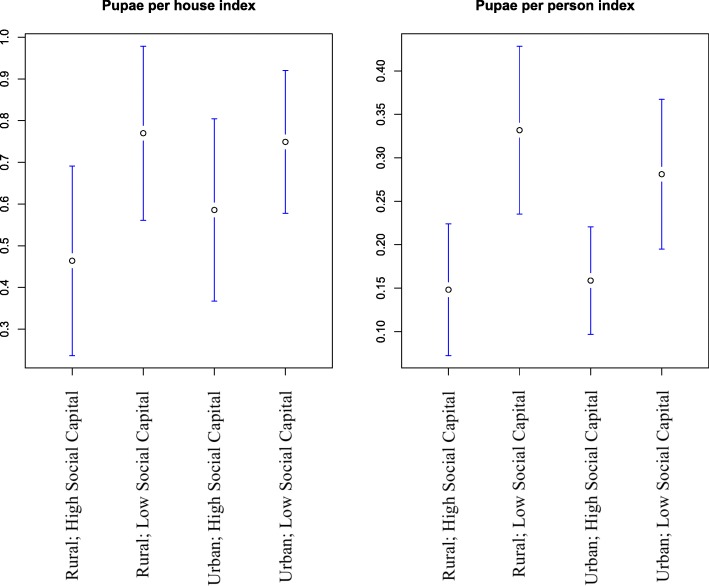
Mean pupal indices with high and low social capital in rural and urban areas. Mean and 95% confidence interval for each index in rural and urban areas for different levels of social capital

The mean PHI and the mean PPI were lower among households with a high social capital score. This difference was only significant among households in rural areas (Fig. 3).

Tables 5 and 6 show the final models of multivariate analyses using the Mantel-Haenszel procedure. When the composite variable of intervention and social capital was included, the independent association between social capital and vector indices did not remain in the final models. The strongest associations in the final models were with the combined intervention/social capital variable and with intervention status.

**Table 5:** Final model of multivariate analysis of associations with absence of larvae or pupae in households

Variable	Crude OR	Adjusted OR	95% CIca adjusted OR
In intervention cluster	1.31	1.45	1.05–2.01
In intervention cluster and high social capital	1.62	1.56	1.19–2.04
Receiving govt social programme	1.29	1.28	1.07–1.53
Low education of household head	0.88	0.83	0.73–0.95
No temephos in household water	0.46	0.41	0.32–0.54

*OR* odds ratio, *CIca* cluster adjusted confidence intervals

**Table 6:** Final model of multivariate analysis of associations with absence of pupae in household

Variable	Crude OR	Adjusted OR	95% CIca adjusted OR
In intervention cluster	1.40	1.50	1.00–2.20
In intervention cluster and high social capital	1.60	1.65	1.20–2.28
Low education of household head	0.72	0.71	0.58–0.87
No temephos in household water	0.43	0.39	0.28–0.52

*OR* odds ratio, *CIca* cluster adjusted confidence intervals

## Discussion

### The social capital index and vector indices

We found a significant association between a high level of social capital and low dengue vector indices. Apart from one small study from Indonesia reporting on correlations between elements of social capital and vector indices [36], we believe this is the first study to examine the association between a measure of social capital and *Aedes aegypti* entomological indices. From this cross-sectional study, we do not know if this is a causal association. If it is causal, a possible mechanism is that a high level of social capital facilitates the transfer of knowledge between community members about how to control *Aedes aegypti* breeding sites. If community members feel that investment of time in community activities, such as mosquito control, is worthwhile, then perhaps they are more likely to participate in such activities. Households that are more involved in their communities may also be more active in eliminating mosquito breeding sites in and around their own living areas.

The social capital index we generated through factor analysis had four constructs: involvement, participation, investment, and communication. These differ somewhat from the four constructs proposed by Siegler [5]; social capital is likely to have different components in different settings. The initial grouping of our 21 questions on social capital into the categories of Siegler and their final grouping from the factor analysis is shown in Fig. 1. Differences between general measures of social capital and our measure are not surprising; as well as general questions about aspects of social capital, we included questions specific to mosquito control for dengue prevention.

The major contributing constructs in our social capital index were participation and involvement, which together explained 35% of the variance of the index. *Participation* reflects the extent to which people, voluntarily or because of certain persuasions or incentives, agree to collaborate on a project, often contributing their work and other resources in exchange for an expected benefit [47]. *Involvement* goes further and reflects the extent to which community members identify and resolve problems in the community on their own terms, in an autonomous, sustainable way [48]. Nelson and Wright define involvement as participation with a purpose, where the community or group establishes a process to control its own development [49].

The *investment* construct in our social capital index is specific to dengue and reflects the benefits individuals or groups expect to get from investing time and money to eliminate mosquito breeding grounds in their homes. Similarly, while communication with family, friends, and community could be a component of a general social capital index, in this study, the *communication* construct of the social capital index reflected communication specifically about how to avoid mosquitoes.

Our finding of higher social capital scores among households in rural sites compared with those in urban sites is consistent with earlier reports from Australia and the USA [50, 51]. The level of social connections between young people may be higher in rural than in urban settings [52].

### The *Camino Verde* intervention

The data in this secondary analysis came from the impact survey of the Camino Verde trial. The trial demonstrated a convincing reduction in vector indices in intervention clusters compared with control clusters [33]. We intended this secondary analysis to shed light on possible mechanisms by which the intervention reduced vector indices. Our current analysis does not support the idea that the impact of the intervention was mediated through increases in social capital. Social capital was not convincingly higher in intervention sites compared with control sites in the impact survey. Further research designed to measure social capital before and after community mobilisation interventions such as Camino Verde could help to answer this question.

Our findings provide some support for the notion that the *Camino Verde* intervention interacted positively with existing patterns of social capital to reduce vector indices. There was a significant interaction between the intervention and social capital in the multivariate analysis. The households least likely to have immature forms of the dengue vector present were those with high social capital located in intervention communities.

In the *Camino Verde* intervention, each intervention community designed its own set of actions to prevent dengue; a discussion of local evidence led to a local action plan for control of the vector. The programme facilitators encouraged community members to plan their own actions to control the dengue vector and thus reduce the risk of dengue, based on the evidence about the mosquito life cycle and breeding grounds in their communities. We believe this evidence-based, participatory approach is an important reason for the success of the intervention [53–55]. A possible explanation for the interaction between social capital and the intervention that we demonstrated in this analysis could be that households with high social capital were more likely to participate in the evidence-based discussions about mosquito-control in the intervention communities, and these discussions then gave them the necessary knowledge to guide their activities to control *Aedes aegypti* breeding sites. Intervention studies that measure social capital prospectively could explore this possibility.

Our findings make a modest contribution to the wider discussion about social capital and health. Studies have reported associations between different aspects of social capital and health [56–58] and there is evidence that some of this association might be mediated by health behaviours [59, 60]. Most of the reported studies are observational, making it difficult to disentangle causality. There are few interventional studies that seek to change social capital and measure the impact on health behaviours and health [61]. The *Camino Verde* trial of community mobilisation for dengue prevention seems to have changed health behaviour (reducing mosquito breeding sites) at least partly through an interaction with social capital, even if it did not change social capital. This could be a useful starting point for future research.

### Limitations

The 21 questions that we included in the factor analysis to create our social capital index might have missed aspects of social capital that are relevant to control of the dengue vector and are amenable to change in intervention sites, particularly in urban areas. We consider our factor analysis to develop the social capital index was robust and benefited from considering three criteria, rather than only a scree plot. The consistency of the index created using the three measures was reassuring. Some questions in our social capital index were specific to dengue prevention and mosquito control. Our index might be applicable in other studies of social capital and dengue prevention, but it would need to be modified for measuring social capital in other circumstances.

## Conclusion

This is the first published study to demonstrate an association between a high social capital score and lower dengue vector indices. Our findings suggest interaction between a community mobilisation intervention and social capital in reducing vector indices and this merits further examination. Understanding such interactions may help to maximise the impact of future community mobilisation interventions.

## Supplementary information

**Additional file 1:.** Sensitivity analyses of different constructions of the social capital score. Table S1. Index range and differential categorization of households into low and high social capital, for four different scenarios of construction of the index. Table S2. Associations between social capital and absence of larvae/pupae and of pupae for the four different constructions of the social capital score

## Data Availability

The datasets used and/or analysed during the current study are available from the corresponding author on reasonable request.

## Abbreviations

95% CIca: Cluster adjusted 95% confidence interval
PPI: Pupae per person index
PHI: Pupae per household index

## References

[1] Social Capital Research: a comprehensive resource on social capital and its research. http://www.socialcapitalresearch.com/definition.html. Accessed 29 Dec 2017.

[2] Bourdieu P. The forms of capital. Handbook of theory and research for the sociology of education. New York: Greenwood; 1986:241-58.

[3] Coleman JS (1990). Foundations of social theory.

[4] Putnam R (2000). Bowling alone: the collapse and revival of American community.

[5] Siegler V. Measuring social capital. Office for National Statistics. UK, 2014. http://webarchive.nationalarchives.gov.uk/20160105213847/http://www.ons.gov.uk/o?A3B2ns/rel/wellbeing/measuring-national-well-being/measuring-social-capital--july-2014/art-measuring-social-capital.html. Accessed 29 Dec 2017

[6] Agampodi TC, Agampodi SB, Glozier N, Siribaddana S (2015). Measurement of social capital in relation to health in low and middle income countries (LMIC): a systematic review. Social Science & Medicine..

[7] Acquaah M, Amoako-Gyampah K, Gray B, Nyathi NQ. Measuring and valuing social capital: a systematic review. Network for Business Sustainability. 2015; https://nbs.net/p/measuring-amp-valuing-social-capital-6fc98c3a-308a-4073-8b9a-7a8341e2d43f. .

[8] Ehsan AM, De Silva MJ. Social capital and common mental disorder: a systematic review. J Epidemiol Community Health. 2015;0:1-8. doi:1136/jech-2015-205868. 10.1136/jech-2015-205868 26179447

[9] Office for National Statistics. Measuring national well-being: Life in the UK, Apr 2017. https://www.ons.gov.uk/peoplepopulationandcommunity/wellbeing/articles/measuringnationalwellbeing/apr2017. Accessed 29 Dec 2017.

[10] Berkman LF, Glass T, Brissette I, Seeman TE (2000). From social integration to health: Durkheim in the new millennium. Soc Sci Med..

[11] Kawachi I, Kennedy BP, Glass R (1999). Social capital and self-rated health: a contextual analysis. Am J Public Health..

[12] Yen IH, Kaplan GA (1999). Neighborhood social environment and risk of death: multilevel evidence from the Alameda County Study. Am J Epidemiol..

[13] Frank L, Kavage S, Litman T. Promoting public health through smart growth: building healthier communities through transportation and land use policies and practices. Smart Growth BC. 2006; http://vtpi.org/sgbc_health.pdf. .

[14] Sampson RJ, Raudenbush SW, Earls F (1997). Neighborhoods and violent crime: a multilevel study of collective efficacy. Science..

[15] Steptoe A, Feldman PJ (2001). Neighborhood problems as sources of chronic stress: development of a measure of neighborhood problems, and associations with socioeconomic status and health. Ann Behav Med..

[16] Drukker M, Buka SL, Kaplan C, McKenzie K, Van Os J (2005). Social capital and young adolescents’ perceived health in different sociocultural settings. Soc Sci Med..

[17] Cramm JM, Van Dijk HM, Nieboer AP (2013). The importance of neighborhood social cohesion and social capital for the well being of older adults in the community. The Gerontologist..

[18] Wakefield SE, Elliott SJ, Cole DC, Eyles JD (2001). Environmental risk and (re) action: air quality, health, and civic involvement in an urban industrial neighbourhood. Health & Place..

[19] Kawachi I, Takao S (2013). Subramanian SV(Eds.). Global perspectives on social capital and health.

[20] Lomas J (1998). Social capital and health: implications for public health and epidemiology. Soc Sci Med..

[21] Kennedy BP, Kawachi I, Brainerd E (1998). The role of social capital in the Russian mortality crisis. World Development..

[22] Barnett ED (2007). Yellow fever: epidemiology and prevention. Clin Infect Dis..

[23] World Health Organization. Dengue and severe dengue. 2009. http://www.who.int/mediacentre/factsheets/fs117/en/. Accessed 29 Dec 2017.

[24] Pan American Health Organization, World Health Organization. Scientists studying intensified vector control measures to combat Zika, dengue and chikungunya in the Americas. http://www.paho.org/hq/index.php?option=com_content&view=article&id=11780. .

[25] Jonker EF, Visser LG, Roukens AH (2013). Advances and controversies in yellow fever vaccination. Ther Adv Vaccines..

[26] Schmitz J, Roehrig J, Barret A, Hombach J (2011). Next generation dengue vaccines: a review of candidates in preclinical development. Vaccine..

[27] World Health Organisation. Immunization, vaccines and biologicals: dengue vaccine research. 2016. http://www.who.int/immunization/research/development/dengue_vaccines/en/. Accessed 29 Dec 2017.

[28] Vanlerberghe V, Toledo ME, Rodríguez M. Gomez D, Baly A, Benitez JR, et al. Community involvement in dengue vector control: cluster randomised trial. BMJ. 2009;12(1):41-47. 20387334

[29] Arunachalam N, Kishore Tyagi B, Samuel M, Krishnamoorthi R, Manavalan R, Chandra TS (2012). Community-based control of Aedes aegypti by adoption of eco-health methods in Chennai City. India. Pathogens and Global Health..

[30] Abeyewickreme W, Wickremasinghe AR, Karunatilake K, Sommerfeld J, Kroger A (2012). Community mobilization and household level waste management for dengue vector control in Gampaha district of Sri Lanka; an intervention study. Pathogens and Global Health..

[31] Castro M, Sánchez L, Pérez D, Carbonell N, Lefèvre P, Vanlerberghe V (2012). A community empowerment strategy embedded in a routine dengue vector control programme: a cluster randomised controlled trial. Trans R Soc Trop Med Hyg..

[32] Caprara A, De Oliveira-Lima J, Rocha-Peixoto A, Vasconcelos-Mota C, Soares-Nobre J, Sommerfeld J (2015). Entomological impact and social participation in dengue control: a cluster randomized trial in Fortaleza, Brazil. Trans R Soc Trop Med Hyg..

[33] Andersson N, Nava-Aguilera E, Arosteguí J, Morales-Perez A, Suaso-Laguna H, Legorreta-Soberanis J (2015). Evidence based community mobilisation for dengue prevention in Nicaragua and Mexico (Camino Verde, the Green Way): cluster randomized controlled trial. BMJ..

[34] Alvarado-Castro V, Paredes-Solís S, Nava-Aguilera E, Morales-Pérez, Alarcón-Morales L, Balderas-Vargas NA, et al. Assessing the effects of interventions for *Aedes aegypti* control: Systematic review and meta-analysis of cluster randomised controlled trials. BMC Public Health. 2017;17(Suppl 1):384. 10.1186/s12889-017-4290-z. 10.1186/s12889-017-4290-z PMC5506587 28699552

[35] Smiljanić J, Mitrović DM (2017). Associative nature of event participation dynamics: a network theory approach. PLoS One..

[36] Subaris H (2016). Subiyanto, Kartono DT, Lestary E. Social capital capacity as prediction of dengue control. International Journal of Public Health Science..

[37] Honaker J, King G, Blackwell M. Amelia II: a program for missing data. https://gking.harvard.edu/amelia. .

[38] Rubin DB. Multiple imputation for non-response in surveys. New York:John Wiley. 1987.

[39] Kabacoff RI (2011). R in action: data analysis and graphics with R.

[40] Core R. Team. R: a language and environment for statistical computing. Vienna: R Foundation for Statistical. Computing. 2015; http://www.R-project.org/. .

[41] Focks DA, Brenner RJ, Hayes J, Daniels E (2000). Transmission thresholds for dengue in terms of Aedes aegypti pupae per person with discussion of their utility in source reduction efforts. Am J Trop Med Hyg..

[42] Focks DA. A review of entomological sampling methods and indicators for dengue vectors. World Health Organization on behalf of the Special Programme for Research and Training in Tropical Diseases., 2004. http://apps.who.int/iris/bitstream/10665/68575/1/TDR_IDE_DEN_03.1.pdf. Accessed 29 Dec 2017.

[43] Andersson N, Mitchell S (2006). Epidemiological geomatics in evaluation of mine risk education in Afghanistan: introducing population weighted raster maps. Int J Health Geogr..

[44] Mantel N, Haenszel W (1959). Statistical aspect of the analysis of data from retrospective studies of disease. J Natl Cancer Inst..

[45] Zelen M (1971). The analysis of several 2x2 contingency tables. Biometrika..

[46] Andersson N, Lamothe G (2011). Clustering and meso-level variables in cross-sectional surveys: an example of food aid during the Bosnian crisis. BMC Health Services Research..

[47] Kahssay HM, Oakley P (1999). Community involvement in health development: a review of the concept and practice.

[48] Morgan LM (2001). Community participation in health: perpetual allure, persistent challenge. Health Policy Plan..

[49] Nelson N, Wright S (1995). Power and participatory development: theory and practice.

[50] Ziersch AM, Baum F, Darmawan IGN, Kavanagh AM, Bentley RJ (2009). Social capital and health in rural and urban communities in South Australia. Australia and New Zealand Journal of Public Health..

[51] Hofferth SL, Iceland J (1998). Social capital in rural and urban communities. Rural Sociology..

[52] Loutfi D, Andersson N, Law S, Salsberg J, Haggerty J, Kgakole L, Cockcroft A (2019). Can social network analysis help to include marginalized young women in structural support programmes in Botswana?. A mixed methods study. International Journal for Equity in Health.

[53] Andersson N (2017). Community-led trials: intervention co-design in a cluster randomised controlled trial. BMC Public Health..

[54] Ledogar RJ, Arosteguí J, Hernández-Alvarez C, Morales-Perez A, Nava-Aguilera E, Legorreta-Soberanis J, Suazo-Laguna H, Belli A, Laucirica J, Coloma J, Harris E, Andersson N (2017). Mobilising communities for Aedes aegypti control: the SEPA approach. BMC Public Health.

[55] Morales-Perez A, Nava-Aguilera E, Legorreta-Soberanis J, Paredes-Solís S, Balanzar-Martínez A, Serrano-de los Santos FR, et al. Which green way: description of the intervention for mobilising against *Aedes aegypti* under difficult security conditions in southern Mexico. BMC Public Health. 2017;17(Suppl 1):398. 10.1186/s12889-017-4300-1. 10.1186/s12889-017-4300-1 PMC5506570 28699562

[56] Murayama H, Fujiwara Y, Kawachi I (2012). Social capital and health: a review of prospective multilevel studies. J Epidemiol.

[57] Ehsan AM, De Silva MJ (2015). Social capital and common mental disorder: a systematic review. J. Epidemiol. Community Health.

[58] Alvarez EC, Kawachi I, Romani JR (2017). Family social capital and health – a systematic review and redirection. Sociology of Health and Illness.

[59] Mohnen SM, Völker B, Flap H, Groenewegen PP (2012). Health-related behavior as a mechanism behind the relationship between neighborhood social capital and individual health - a multilevel analysis. BMC Public Health.

[60] Nieminen T, Prättälä R, Martelin T, Härkänen T, Hyyppä MT, Alanen E, Koskinen S (2013). Social capital, health behaviours and health: a population-based associational study. BMC Public Health.

[61] Villalonga-Olivesa E, Wind TR, Kawachi I (2018). Social capital interventions in public health: a systematic review. Social Science & Medicine.

