# Supplementary material for: Social capital is associated with lower mosquito vector indices: secondary analysis from a cluster randomised controlled trial of community mobilisation for dengue prevention in Mexico
Source: Popul Health Metr. 2019 Dec 10;17:18. doi: 10.1186/s12963-019-0199-3 (PMC6902442; doi:10.1186/s12963-019-0199-3)
Supplement: Supplementary file 1 — Additional file 1:. Sensitivity analyses of different constructions of the social capital score. Table S1. Index range and differential categorization of households into low and high social capital, for four different scenarios of construction of the index. Table S2. Associations between social capital and absence of larvae/pupae and of pupae for the four different constructions of the social capital score [file 12963_2019_199_MOESM1_ESM.docx]

**Supplementary data file 1: sensitivity analyses of different constructions of the social capital score**

We analysed four different scenarios, with different sets of variables in the social capital index, using factor analysis. In each case the social capital index included four constructs. Table S1 shows the minimum and maximum values of the index for the four scenarios, and the percentage of households classified differently into low or high social capital, compared with scenario 1.

Table S1. Index range and differential categorization of households into low and high social capital, for four different scenarios of construction of the index

| Scenario | Minimum value | Maximum value | Percentage of different classification into low and high* |
| --- | --- | --- | --- |
| 1. All 21 variables (21) | -0.77 | 0.84 | N/A |
| 2. 16 variables | -0.76 | 0.88 | 0.9% |
| 3. 14 variables | -0.73 | 0.82 | 1.3% |
| 4. 12 variables | -0.93 | 0.60 | 5.5% |

*Compared with scenario 1

Table S2 shows the OR, 95% CIca, and Mantel-Haenszel chi-square for the associations between high social capital score and absence of larvae and/or pupae and absence of pupae only, for the four scenarios of constructing the social capital score. The values of OR, 95%CIca and Mantel-Haenszel chi-square are stable across the four scenarios for construction of the social capital index.

Table S2. Associations between social capital and absence of larvae/pupae and of pupae for the four different constructions of the social capital score

| High social capital score scenario | Association with being negative for larvae & pupae | | |
| --- | --- | --- | --- |
|  | OR | 95% CIca | Mantel-Haenszel chi-square |
| 1. All variables (21) | 1.38 | 1.12-1.69 | 31.59 |
| 2. 16 variables | 1.39 | 1.13-1.70 | 32.47 |
| 3. 14 variables | 1.41 | 1.15-1.73 | 35.57 |
| 4. 12 variables | 1.40 | 1.15-1.69 | 34.18 |
| High social capital score scenario | Association with being negative for pupae | | |
|  | OR | 95% CIca | Mantel-Haenszel estimate |
| 1. All variables (21) | 1.37 | 1.08-1.74 | 17.12 |
| 2. 16 variables | 1.37 | 1.07-1.74 | 17.09 |
| 3. 14 variables | 1.36 | 1.07-1.74 | 16.72 |
| 4. 12 variables | 1.38 | 1.10-1.74 | 18.08 |
